# Regionalization of cell types in silk glands of *Larinioides sclopetarius* suggest that spider silk fibers are complex layered structures

**DOI:** 10.1038/s41598-023-49587-z

**Published:** 2023-12-14

**Authors:** Sumalata Sonavane, Per Westermark, Anna Rising, Lena Holm

**Affiliations:** 1 Department of Anatomy, Physiology and Biochemistry, Swedish University of Agricultural Sciences, Uppsala, Sweden; 2 Department of Immunology, Genetics and Pathology, Uppsala University, Uppsala, Sweden; 3 Department of Biosciences and Nutrition, Karolinska Institutet, Neo, Huddinge, Sweden

**Keywords:** Cell biology, Anatomy

## Abstract

In order to produce artificial silk fibers with properties that match the native spider silk we likely need to closely mimic the spinning process as well as fiber architecture and composition. To increase our understanding of the structure and function of the different silk glands of the orb weaver *Larinioides sclopetarius*, we used resin sections for detailed morphology, paraffin embedded sections for a variety of different histological stainings, and a histochemical method for localization of carbonic anhydrase activity. Our results show that all silk glands, except the tubuliform glands, are composed of two or more columnar epithelial cell types, some of which have not been described previously. We observed distinct regionalization of the cell types indicating sequential addition of secretory products during silk formation. This means that the major ampullate, minor ampullate, aciniform type II, and piriform silk fibers most likely are layered and that each layer has a specific composition. Furthermore, a substance that stains positive for polysaccharides may be added to the silk in all glands except in the type I aciniform glands. Active carbonic anhydrase was found in all silk glands and/or ducts except in the type I aciniform and tubuliform glands, with the strongest staining in aggregate glands and their ductal nodules. Carbonic anhydrase plays an important role in the generation of a pH gradient in the major ampullate glands, and our results suggest that some other glands may also harbor pH gradients.

## Introduction

Spider silk fibers have unique mechanical and physical properties which make them interesting for applications in medicine and industry^1^. However, mass production of native silk using spiders is laborious and far from economically feasible since, unlike silkworms, individual spiders produce small amounts of silk. It is also challenging to breed spiders due to their territorial and cannibalistic nature. Alternative methods of producing silk by recombinant technologies offer promising solutions to these challenges and are being developed^2–4^. However, in order to truly mimic the pristine spider silk fiber, we need to develop better spinning methods, and we believe that the best way to do so is to characterize the spiders’ spinning apparatus, which hopefully can give us clues for the design of novel biomimetic spinning devices as well as to increase our understanding of the composition and architecture of the silk fiber.

Swedish bridge spiders (*Larinioides sclopetarius*) were chosen for this study since they belong to the family Araneidae which means that the females can spin seven types of silk. The silks are produced in glands located in the opisthosoma and include the major ampullate, minor ampullate, flagelliform (or coronate), aggregate, piriform, tubuliform (or cylindrical), and aciniform glands. Each silk type has its characteristic structural, mechanical, and functional properties, and the silk glands that process them have distinct morphologies^5–12^. The major ampullate gland is the most well-studied silk gland and is composed of a tail where the main synthesis of the spidroins takes place, a wider sac where the proteins are stored, and an S-shaped duct where the soluble spidroins are converted into a fiber^13^. There are a number of studies on the histology and ultrastructural morphology of the major ampullate glands^5,11, 14–19^, and one of these have shown that the tail and the sac are made of three types of epithelial cells confined to three zones (A–C)^11^. Silk polymerization in the major ampullate glands occurs in response to changes in several factors such as decreased pH^20,21^, changes in ion concentrations^22^ as well as shear stress^23^. Several studies have suggested a skin–core architecture for the major ampullate silk^11,14, 24–28^, in which the core contains spider silk proteins (spidroins)^28,29^ whereas the skin layer consists of carbohydrates, specifically glycogen/glycoproteins^24,28^. Others have further suggested a four-layer^30^ and a five-layer^28^ model for the major ampullate silk. The other silk glands have not been studied extensively, but a few investigations exist^5,15, 31–38^.

In the major ampullate gland, carbonic anhydrase (CA) is instrumental in upholding a pH gradient (from 7.6 to < 5.7 halfway through the duct)^21^. This pH gradient is one of the most important factors that regulate spidroin solubility and polymerization. CA is present in other silk-spinning species, e.g. the silkworm *Bombyx mori*, where it has been shown to have a similar function as in the spiders, i.e. generating protons that lower the pH along the silk gland^39^. CAs are a group of metalloenzymes that catalyze the reversible reaction –

$${CO}_{2}+ {H}_{2}O \rightleftharpoons {HCO}_{3}^{-}+ {H}^{+}$$

These enzymes are ubiquitous in all kingdoms and eight genetically distinct CA families have been identified to date: α-, β-, γ-, δ-, ζ-, η-, θ-, and ι-CAs^40,41^. α-CAs are the most well-characterized and are present in several prokaryotic and eukaryotic organisms. CAs perform a variety of functions, i.e. in mammals CA is present in e.g., gastric mucosa where it is involved in acid secretion and kidney tubules where it regulates acid–base balance and water homeostasis^42–44^.

This study aimed to identify all silk glands of *L. sclopetarius* and describe their morphological features using various methods. Resin-embedded tissue was used to provide good histological detail and a previously described histochemical method^45^ was used to describe the localization of active CA in the glands. Thioflavin-S and Congo red were used to detect amyloid-like fibrils within silk glands, and periodic acid-Schiff (PAS) stain to detect the origin of carbohydrates. Since depletion of silk stored in the sac may result in morphological changes of the major ampullate gland^46^, one group of spiders was stimulated to produce silk before sacrifice and the histological appearance of the major ampullate glands was compared.

## Results

### Morphology of silk glands

**Figure 1:**
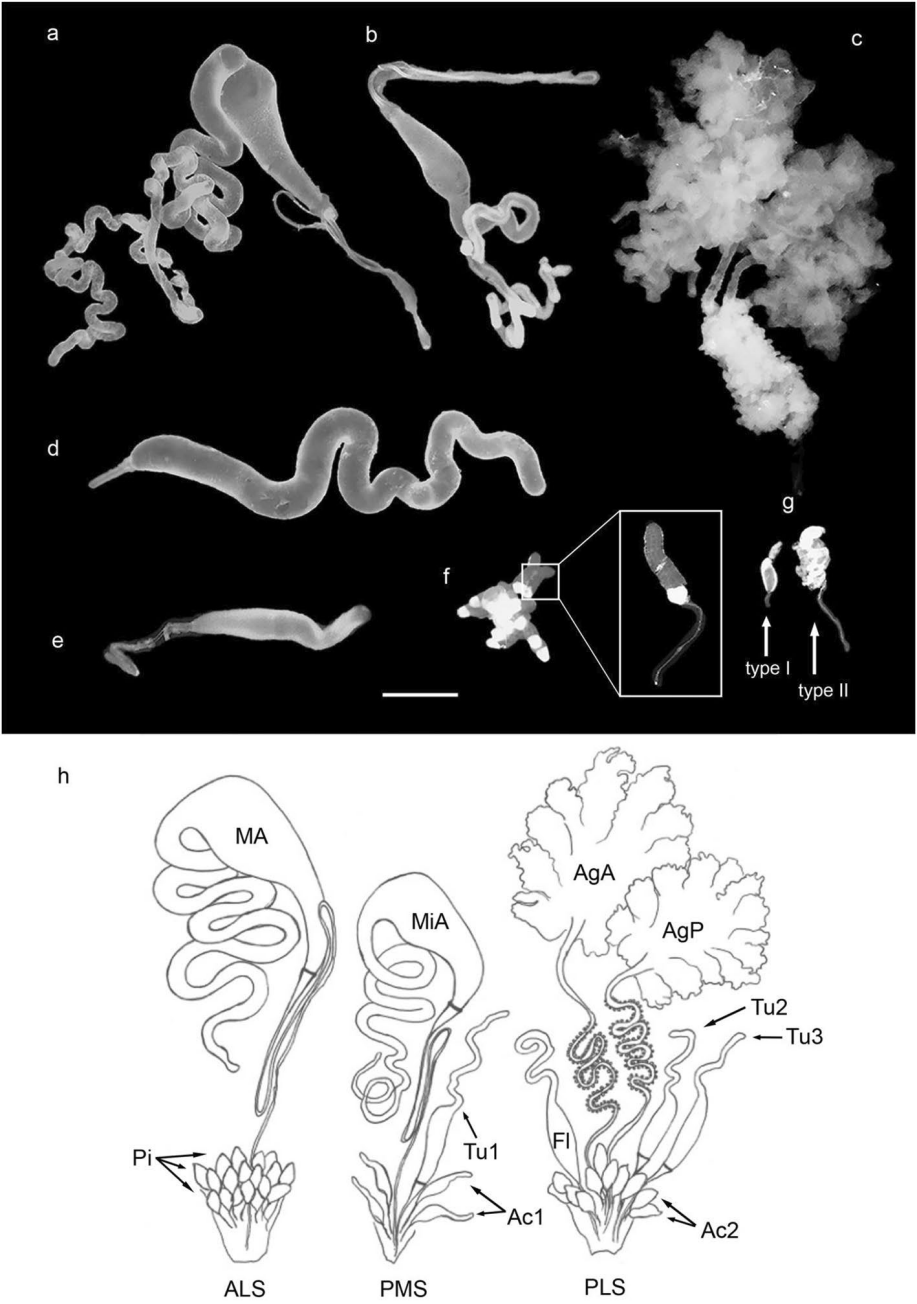
Silk glands of *L. sclopetarius*, only one set of the paired glands is shown for clarity. (**a**) Major ampullate gland*.* (**b**) Minor ampullate gland. (**c**) A pair of aggregate glands. (**d**) Tubuliform gland (from a spider with many mature eggs), only one of the three tubuliform glands from one side is shown. (**e**) Flagelliform gland. (**f**) A group of piriform glands, inset shows magnified image of single gland. (**g**) Aciniform glands (type I & type II). Scale bar in (**a–g**) = 1 mm. Parts of the minor ampullate & flagelliform gland tails were lost during dissection. (**h**) Schematic drawing of silk glands from one side of the opisthosoma in *L. sclopetarius* with an indication of which spinneret the gland is attached to (ALS- anterior lateral spinneret, PMS- posterior median spinneret, PLS- posterior lateral spinneret). MA- major ampullate, MiA- minor ampullate, Pi- group of piriform glands, Tu1–3- tubuliform glands, Fl- flagelliform gland, Ac1, Ac2- aciniform type I and type II glands, AgA- anterior aggregate gland, AgP- posterior aggregate gland).

**Figure 2:**
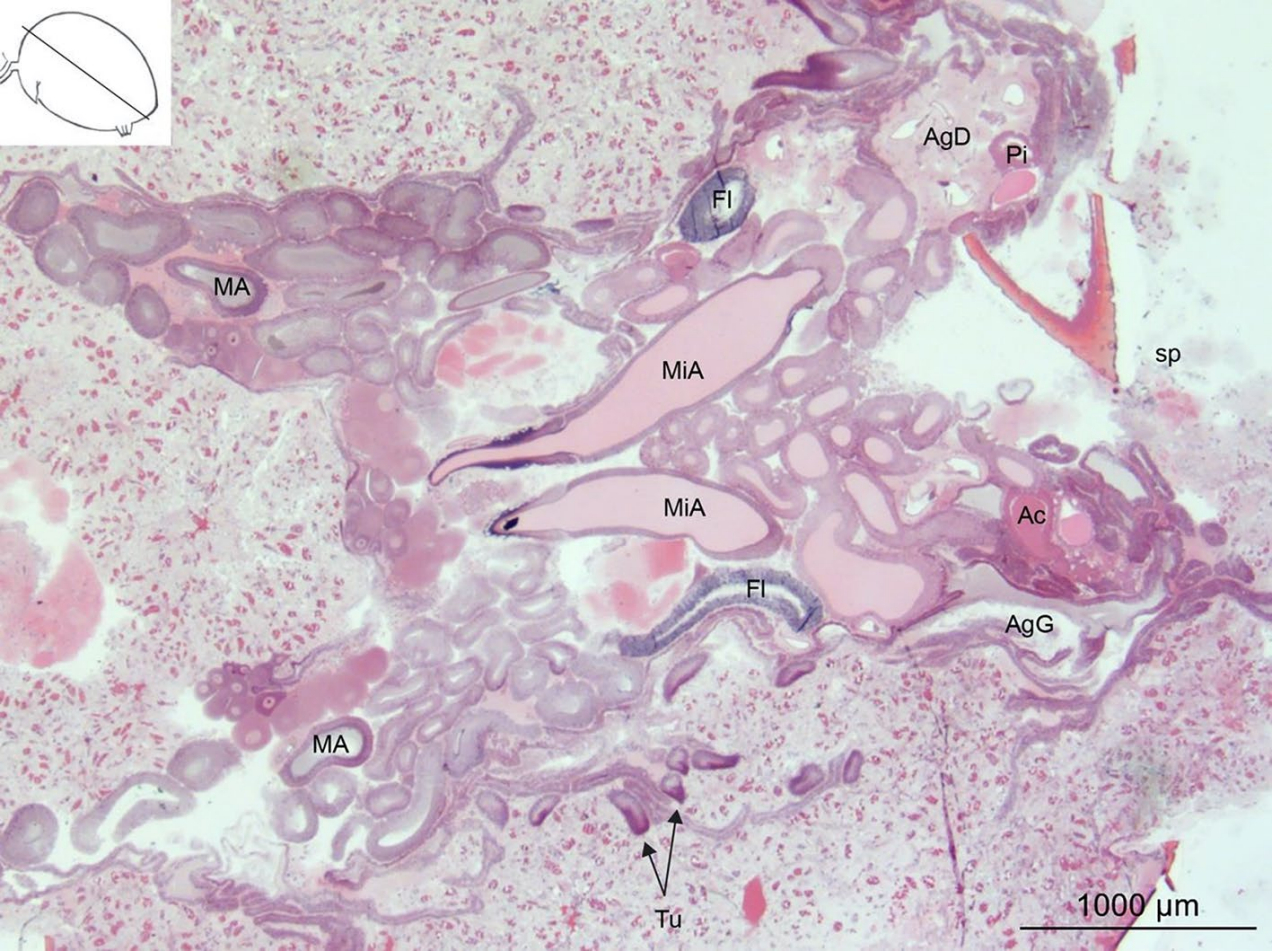
Frontal section of the opisthosoma of an adult female *L. sclopetarius* showing the localization of different glands. *MA* major ampullate, *MiA* minor ampullate, *Fl* flagelliform, *AgG* aggregate gland, *AgD* aggregate duct, *Ac* aciniform, *Tu* tubuliform, *Pi* piriform, *sp* spinnerets. The line in the inset indicates the approximate plane of the section in the opisthosoma. HE.

Seven types of silk glands were identified and could be isolated from the opisthosoma of *L. sclopetarius* (Fig. 1). Figure 2 shows a section of the opisthosoma stained with hematoxylin–eosin (HE) with the location of all the glands indicated.

#### Ampullate glands

Two primary major ampullate glands were located in the opisthosoma, close to the ventral wall. One pair of secondary major ampullate glands that are much smaller in size was attached to the duct of each major ampullate gland (Supplementary, SI Fig. 1). Two minor ampullate glands were located alongside the median plane of the opisthosoma and dorsal to the major glands. The ampullate glands were each composed of a narrow long tail, a wide bulged ampulla, and an S-shaped duct, however, the minor glands were smaller in size than the major glands (Fig. 1a and b). The sac and the duct of ampullate glands were bridged by a structure known as the funnel. The ducts of major glands were connected to the anterior lateral spinneret whereas those of the minor glands were connected to the posterior median spinnerets (Fig. 1h).

**Figure 3:**
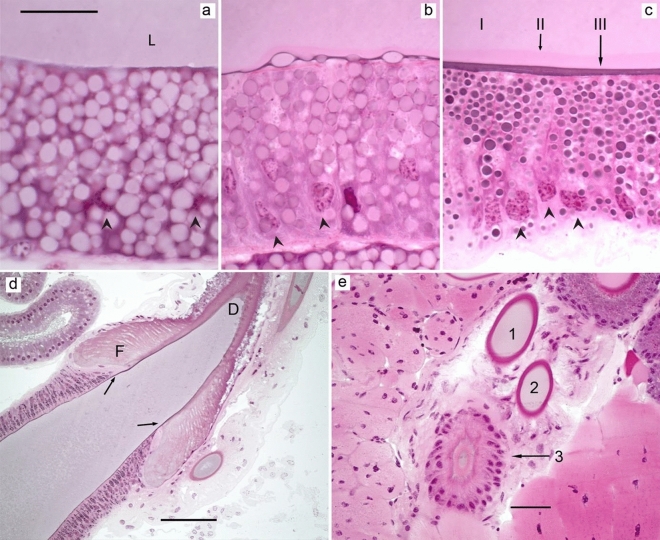
Major ampullate glands of *L. sclopetarius*. (**a**–**c**) Epithelial cells belonging to the three zones A, B and C, respectively, in the tail and sac of the major ampullate gland. The arrowheads indicate basally located nuclei. In (**c**), the three layers in the lumen (indicated as I, II and III), added by the three cell types A, B and C, respectively, can be distinguished. (**d**) Funnel region (F), the duct (D) that starts from the funnel is marked by arrows. (**e**) Cross section of the duct, the three limbs (numbered 1–3) are arranged in a triad fashion showing the decreasing diameter of the duct. Note that the cells surrounding the duct are taller in the third limb. L- lumen. HE, scale bars (**a–c**) = 25 μm, (**d**) = 100 μm and (**e**) = 50 μm.

**Figure 4:**
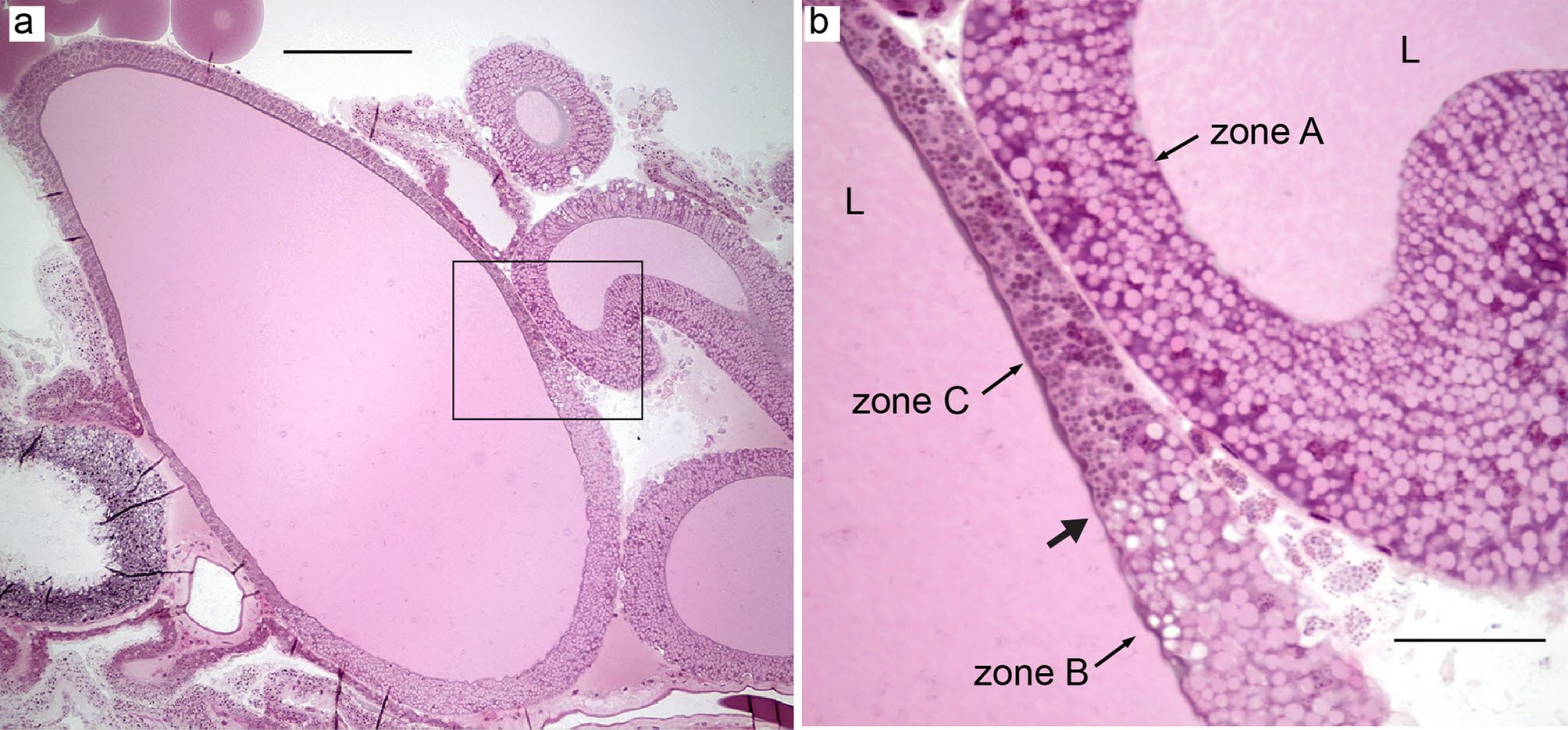
(**a**) Minor ampullate gland of *L. sclopetarius.* The inset is magnified in (**b**). Zones A–C are indicated by thin arrows, thick arrow shows the transition from zone B to C. L- lumen. HE, scale bars (**a**) = 200 μm, (**b**) = 50 μm.

The tail and sac of ampullate glands were composed of three different zones as determined by the appearance of the epithelial cells by cell histology (Figs. 3a–c and 4a,b). The epithelium in the entire tail and the proximal third of the sac was identified as zone A, the central region of the sac as zone B, and the distal end of the sac until the funnel as zone C. The borders between the zones were sharp (Fig. 4b, Supplementary, SI Fig. 2). All zones were made of simple columnar tall epithelial cells with basally located nuclei. The cells of zone A contained big irregular granules that stained weakly with HE. In zone B, the cellular granules were somewhat smaller and stained lightly pink. In zone C, the granules were even smaller and stained intensely with HE. The secretory contents of the three zones stained similar to the granules of the corresponding cells. The secretions seemed to be sequentially added and formed separate layers in the lumen (Fig. 3a–c). The funnel was made of comb-like structures surrounding the lumen and was lined by a flat layer of cells on the basal side. The lumen of the duct was lined with a cuticle (shown by arrows in Fig. 3d) that originated from the funnel and continued till the spinnerets. The diameter of the duct decreased as it approached the spinnerets while the height of the surrounding epithelial cells increased, which was evident from a cross-section of the three limbs arranged in a *triad* fashion (Fig. 3e). Although the secondary major ampullate glands were seen macroscopically (Supplementary, SI Fig. 1), they could not be identified histologically. No histological differences were observed between the major ampullate glands of silked and unsilked spiders.

#### Aggregate glands

Two pairs of aggregate glands, anterior and posterior, were identified. The gland was branched (Fig. 1c,h) and spread across most of the anterior opisthosoma. The ducts were wide, partially convoluted and connected to the posterior lateral spinnerets. The convoluted part of the ducts was surrounded by numerous small nodules (Fig. 1c,h).

**Figure 5:**
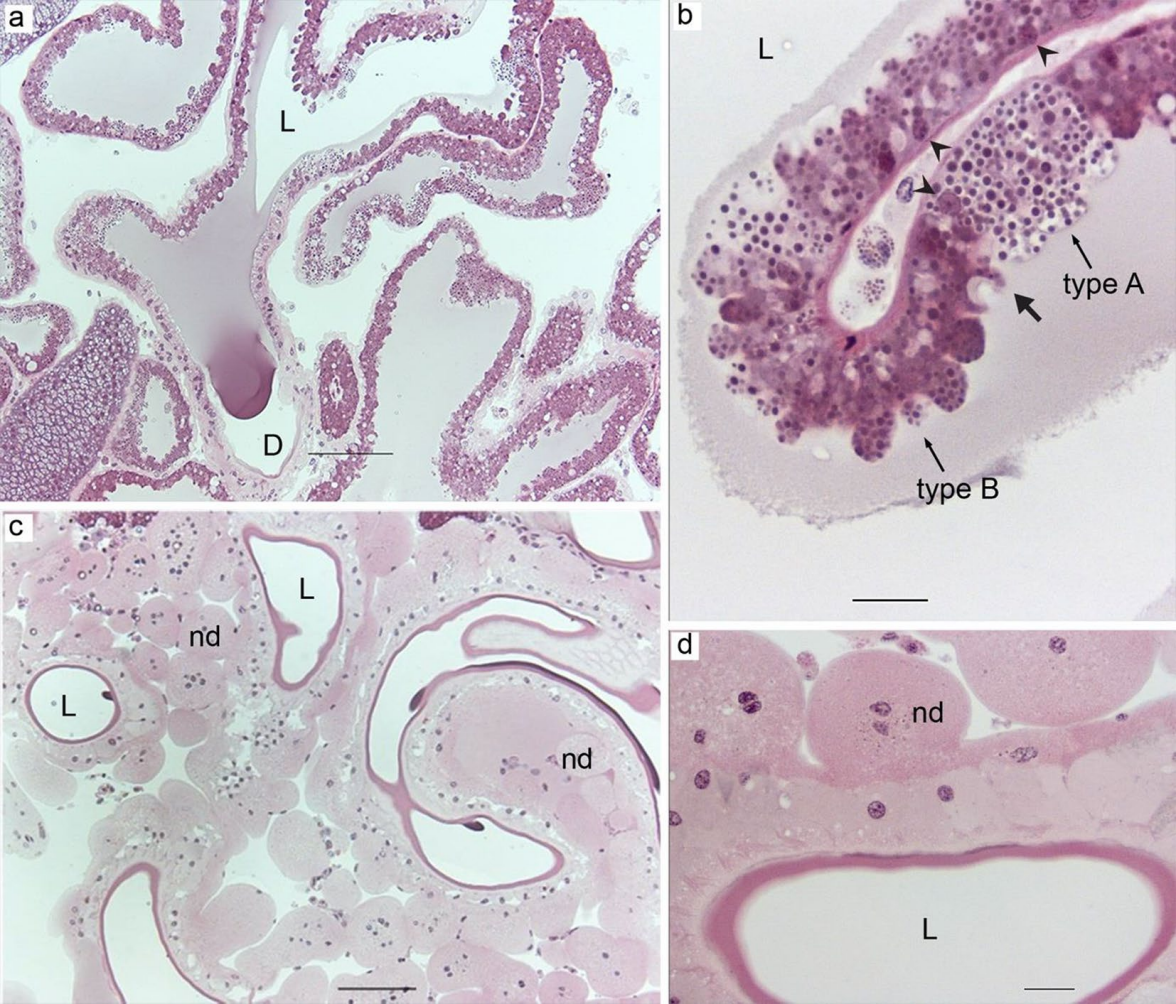
(**a**) Aggregate glands of *L. sclopetarius* showing a thin epithelium and spacious lumen (L), D-duct. (**b**) Two cell types (A & B) and the apical projections containing several granules. Thick arrow indicates the sharp transition between two cell types and the arrowheads indicate nuclei. (**c**) Ducts of aggregate glands. The cuticular intima is surrounded by a single layered epithelium. The ducts have numerous nodules (nd). (**d**) Magnified image of a duct showing the cells surrounding the cuticular intima and the cells of the nodules. HE, scale bar (**a**,**c**) = 100 μm and in (**b**,**d**) = 20 μm.

Aggregate glands were branched and characterized by a spacious lumen (Fig. 5a). The epithelium consisted of two types of cells (type A and B) (Fig. 5b), both simple columnar cells with basally located oval-shaped nuclei. Type A cells were tall and contained somewhat irregular granules that stained intensely with HE. Type B cells were slightly shorter, more abundant, and had heterogeneous granules. In addition to intensely staining granules similar to type A cells, they had two other types of granules that stained pink and light pink. The border between the two cell types was distinct, but they could not be confined to specific zones. Apically the cells were irregular and had bulging structures filled with numerous granules (Fig. 5b). The ducts of aggregate glands were composed of a cuticular intima that was surrounded by a single layer of cuboidal epithelial cells which was further surrounded by numerous nodules. The nodules were macroscopically visible (Fig. 1c) and consisted of a group of large cells with irregular nuclei and indefinite cell boundaries (Fig. 5c,d). In the opisthosoma, the ducts of two aggregate glands were located along with the duct of one flagelliform gland which can be visualized as a *triad* on the cross-sections (Supplementary, SI Fig. 3), close to the posterior lateral spinneret.

#### Tubuliform glands

**Figure 6:**
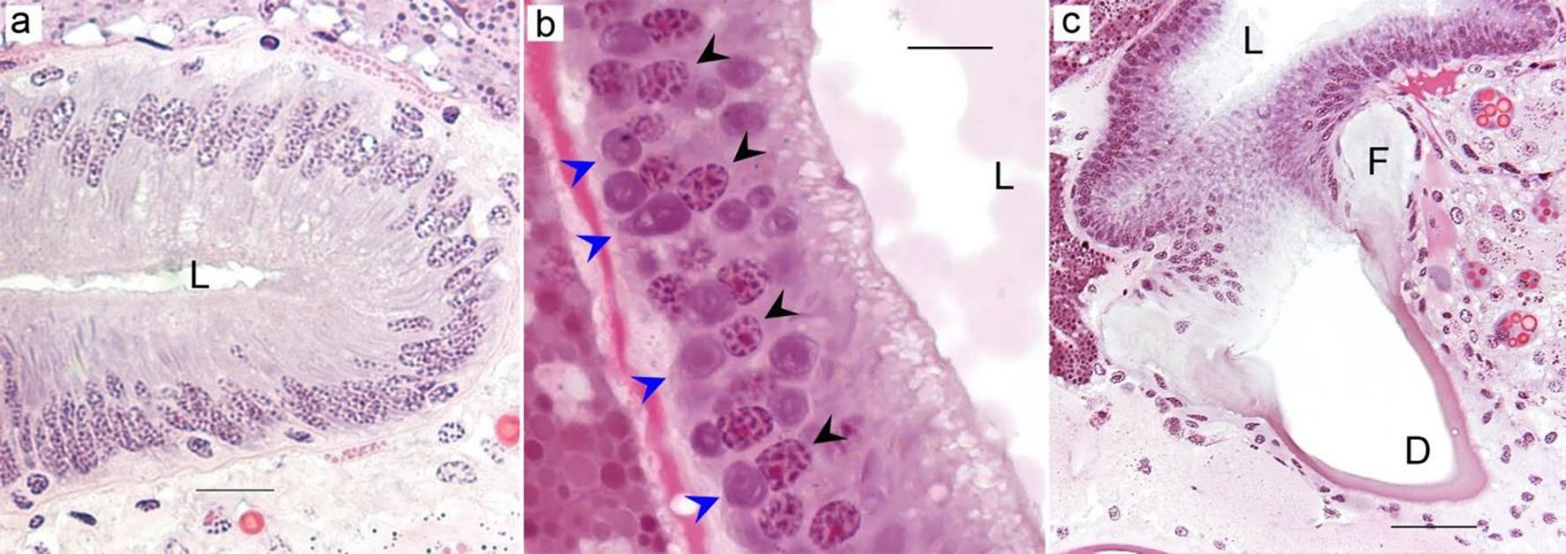
Tubuliform glands from two *L. sclopetarius*. (**a**) Tubuliform gland in female with no mature eggs. The lumen is narrow, cells are tall, and the nuclei are elongated. (**b**) Tubuliform gland in female with many mature eggs. The cells are low columnar with almost round nuclei and the lumen shows secretion. Black arrowheads indicate nuclei and blue arrowheads indicate structures resembling endoplasmic reticulae. (**c**) Funnel that connects the gland to the duct. L-lumen, D-duct. HE, scale bars: (**a**,**b**) = 10 μm, and (**c**) = 50 μm.

Three tubuliform glands were located on each side of the median plane, one was connected to the posterior median spinneret and two were connected to the posterior lateral spinneret (Fig. 1h). These glands were long, tubular, and had thin short ducts (Fig. 1d). The tubular part of the gland narrowed towards the proximal end. The tubuliform glands were composed of simple columnar epithelium but the morphology of these glands varied between individuals. In some spiders, the gland had a single layer of tall columnar epithelial cells with very elongated, basally located nuclei and a constricted lumen (Fig. 6a). In spiders with large eggs, the epithelium was cuboidal or low columnar and nuclei were oval and basally located. In this case, the glandular lumen was very spacious and harbored secretory products that stained light pink with HE (Fig. 6b). The epithelial cells contained several concentric structures close to the nuclei which stained bright pink (Fig. 6b, blue arrowheads). The duct was lined with a cuticle and connected to the gland via a funnel similar to ampullate funnels (Fig. 6c).

#### Aciniform glands

Two types of aciniform glands were located close to the spinnerets. Type I aciniform glands consisted of a sac with a small tail at the proximal end. They occurred singly and their ducts connected to the posterior median spinneret. Type II aciniform glands were pear-shaped and presented in clusters. Their ducts connected to the posterior lateral spinnerets (Fig. 1g, h).

**Figure 7:**
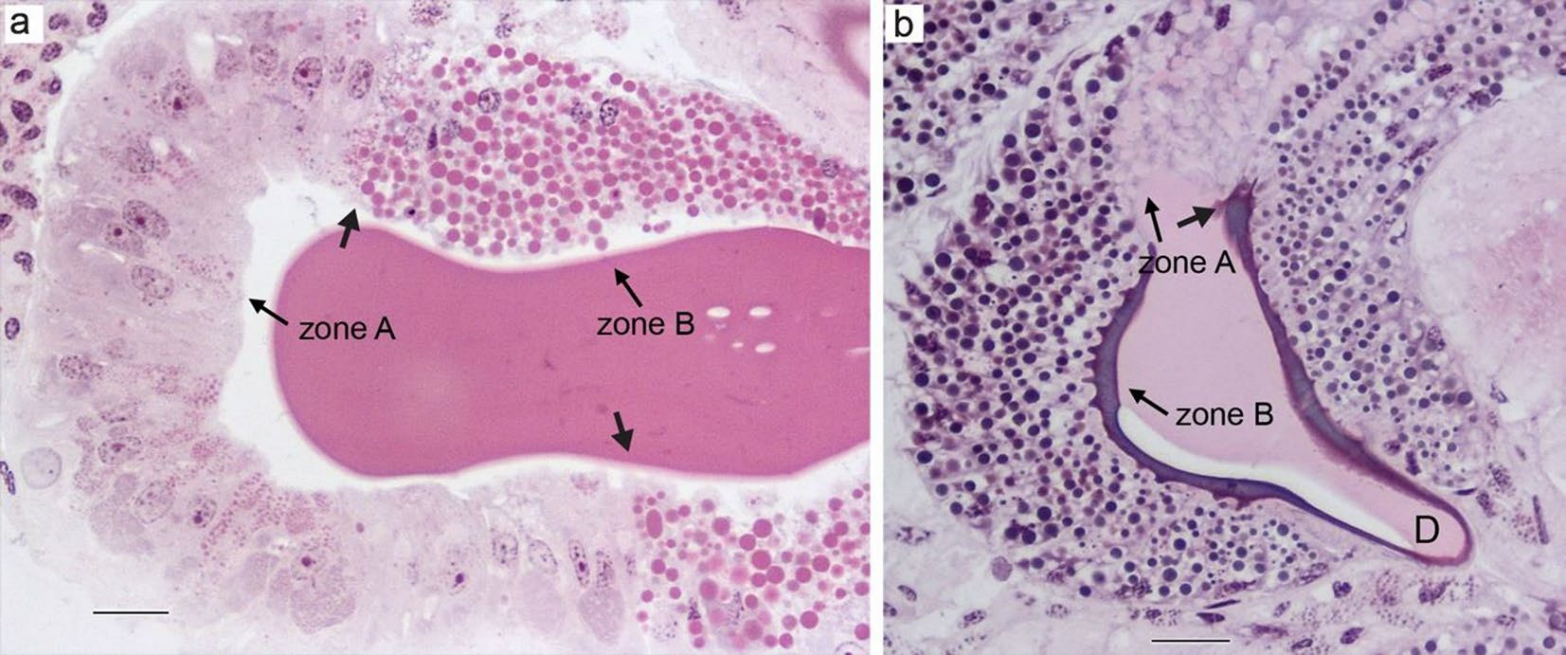
Aciniform glands of *L. sclopetarius*. (**a**) Type I aciniform gland showing two kinds of cells. (**b**) Type II aciniform gland showing two cell types that stain differently compared to type I aciniform cells. Zones are indicated in both types of glands. Thick arrows indicate the sharp transition between the zones, D-duct. HE, scale bars: (**a**) = 50 µm and (**b**) = 20 μm.

Type I aciniform glands were composed of two zones with histologically distinct features. The tail (zone A) had simple columnar epithelial cells with basally located nuclei and their granules stained light pink with HE. The distal part of the gland (zone B) had similar cells, but the granules stained bright pink (Fig. 7a).

The type II glands also consisted of two different regions. The proximal end was termed zone A and the distal end zone B. Both zones were composed of simple columnar epithelial cells with basally located nuclei. In zone A, the cellular granules were big and stained light pink. In zone B the cells contained two types of irregular granules that stained dark pink and bright purple. The secretory contents of the granules in zone B appeared to form an outer layer around the secretory content of zone A cells (Fig. 7b).

#### Piriform glands

**Figure 8:**
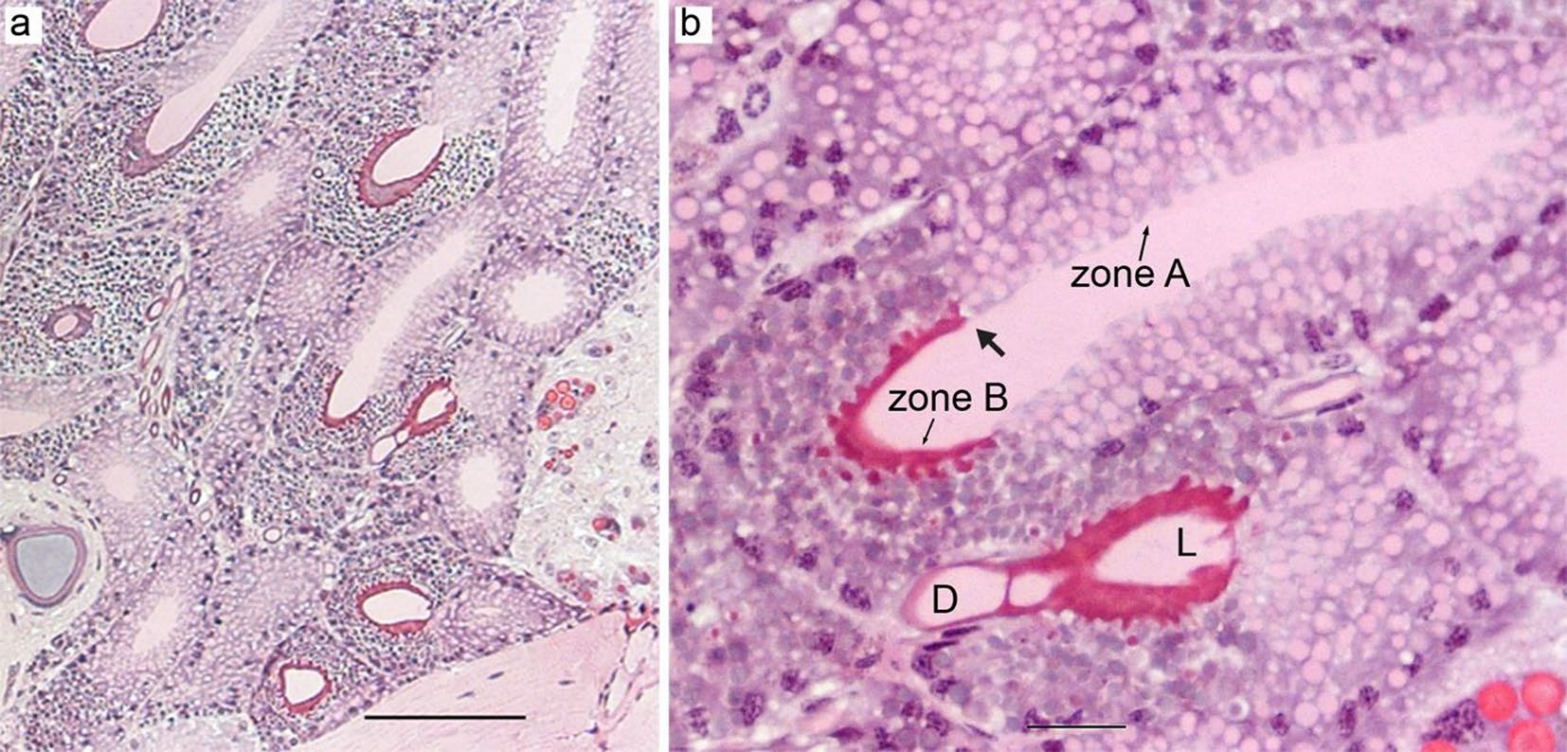
Piriform glands of *L. sclopetarius*. (**a**) A group of piriform glands in the opisthosoma. (**b**) Magnified image of the piriform glands showing two cell types, L-lumen, D-duct. Sharp border between two zones is indicated with an arrow. HE, scale bar: (**a**) = 100 μm, (**b**) = 20 μm.

Several piriform glands were located close to the spinnerets. These glands were macroscopically similar to aciniform type II glands, and their ducts exited through anterior lateral spinnerets (Fig. 1f,h). The gland contained two cellular zones, zone A (proximal end) and zone B (distal end), demarcated with a sharp boundary. In both zones, the cells were composed of a simple columnar epithelium with oval to round-shaped and basally located nuclei. The granules in the cells of zone A were big and stained light pink. In zone B, the cells had small granules which stained red. Zone B was connected to the duct which led to the spinnerets. The secretions of zone B cells formed a layer surrounding the weakly staining bulk content of the gland (Fig. 8).

#### Flagelliform glands

**Figure 9:**
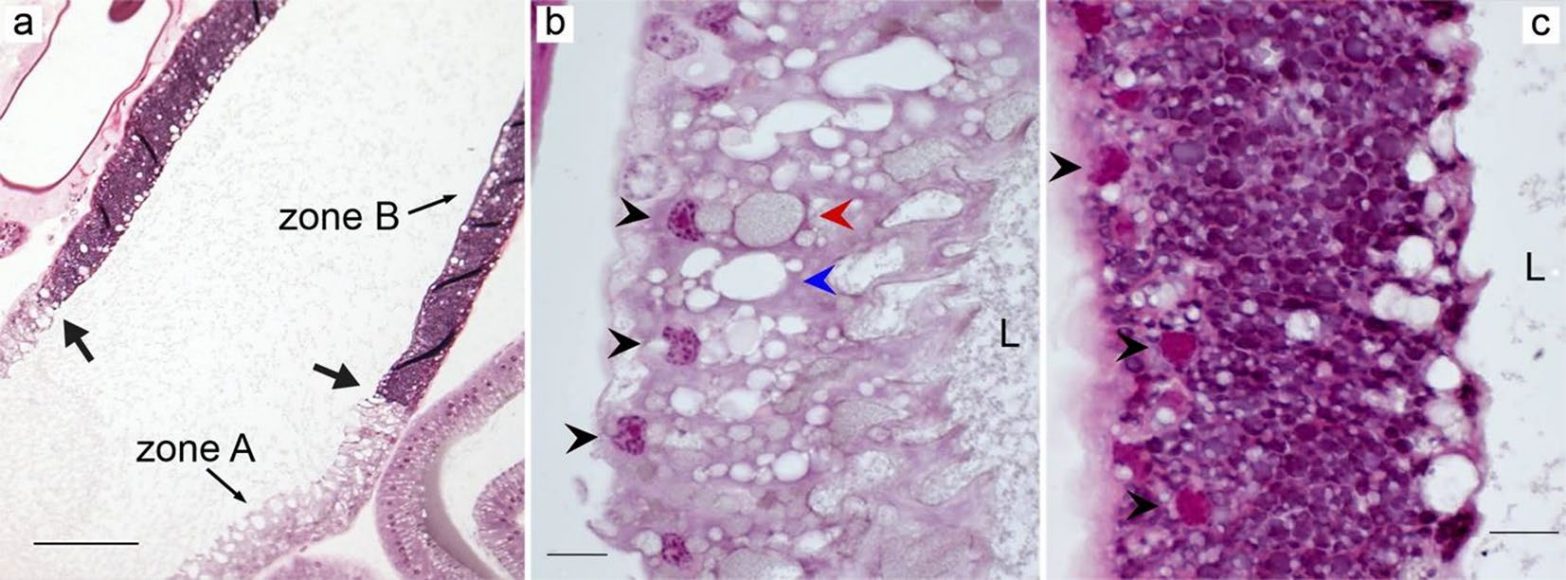
Flagelliform gland from *L. sclopetarius*. (**a**) Arrows indicate transition between zones. (**b**) Zone A cells with weakly stained granules. The apical surface is highly irregular in this region. Nuclei are indicated with black arrowheads. Blue and red arrowheads indicate empty and filled vesicles, respectively. (**c**) Zone B cells showing brightly stained granules and eosinophilic nuclei (black arrowheads), L-lumen. HE, scale bars: (**a**) = 100 μm, (**b**,**c**) = 10 μm.

One pair of flagelliform glands were located dorsally of the minor ampullate glands. In appearance, the glands looked similar to tubuliform glands but had shorter tails and relatively larger sacs compared to their tails (Fig. 1h). We identified two distinct cellular regions, zone A and zone B (Fig. 9). The tail and about half of the sac was termed zone A, whereas the distal part that connected to the duct was termed zone B. Both zones were composed of simple columnar epithelium with basally located nuclei. Zone A cells had spherical nuclei and weakly-staining big, irregular granules. Apically these cells were irregular and appeared to secrete their content in the lumen that stained light pink (Fig. 9b). The cells harbored many granules with different staining content as indicated in Fig. 9b (red and blue arrowheads). Zone B had eosinophilic nuclei and cytoplasm full of evenly sized small secretory granules that stained dark pink (Fig. 9c).

### Carbonic anhydrase activity

During the first step of CA staining, a chemical reaction occurs in locations with active CA whereby CO_2_ leaves, the pH increases, and a cobalt-phosphate-carbonate complex is formed. In the subsequent steps, this complex converts into a black cobalt sulphide precipitate which can be visualized on sections. This method stains all active CA on the sections irrespective of the isoform. In the current study, active CA was found in all glands except the tubuliform glands and type I aciniform glands. Control sections incubated with the CA inhibitor acetazolamide were generally unstained. Low temperature euthanization of spiders produced the same staining results as CO_2_ anesthesia.

**Figure 10:**
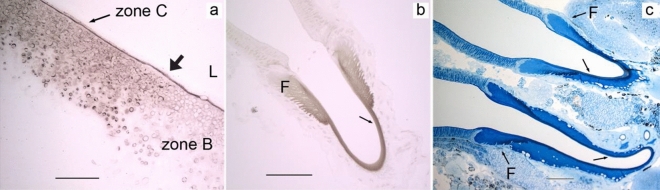
Black staining show carbonic anhydrase activity in the ampullate glands of *L. sclopetarius*. (**a**) Zone C of major ampullate gland showing weak CA activity in granules and apical lining, L- lumen. Arrow indicates the border between zone B & C. (**b**) Funnel (F) and cuticular intima of the major ampullate duct (arrow) show weak CA activity. (**c**) Weak CA activity in zone C, and intermediate in the funnel (F) and cuticle (arrows) of minor ampullate glands. This section was counterstained with azure blue. Scale bars: (**a**) = 50 μm, (**b**) = 100 μm, (**c**) = 20 μm.

In both major and minor ampullate glands, weak to moderate CA activity was observed in granules of the cells in zone C, funnel, and the cuticle of the duct (Fig. 10). No difference was observed in major ampullate glands of silked and unsilked individuals.

**Figure 11:**
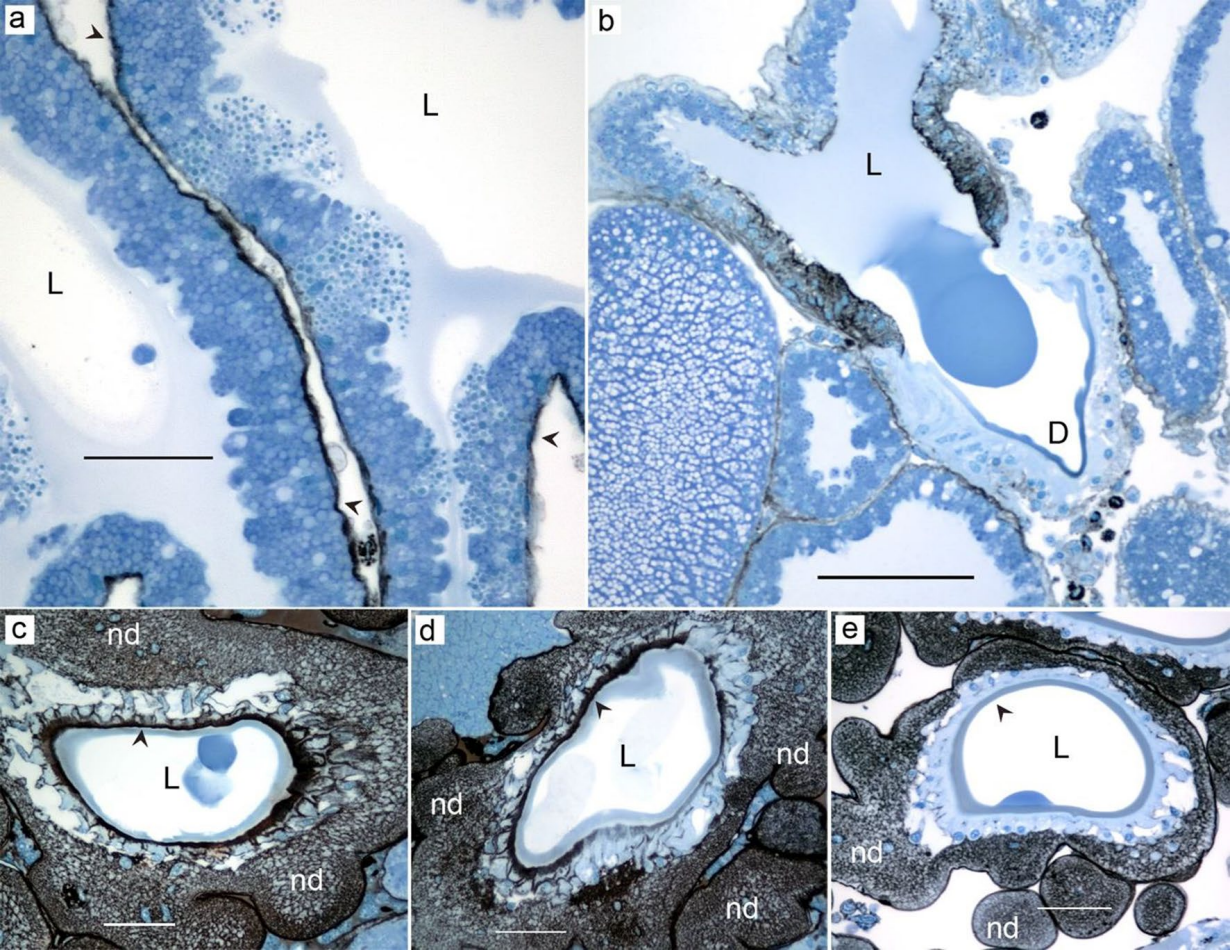
Black staining show carbonic anhydrase activity in aggregate glands of *L. sclopetarius*. (**a**) Active CA is seen throughout the basal membrane of the glands (arrowheads). (**b**) A group of cells close to the duct (D) show active CA. (**c**) Cross-section showing strong staining in cuticular intima of the duct and cell membrane of the surrounding epithelium. (**d**) Cross-section of a duct with variable CA staining. (**e**) Weak staining in the cuticle and no visible staining in the epithelium. In (**c**–**e**), cuticle is indicated with arrowheads. Note the strong staining for CA in the nodules (nd). L-lumen. Azure blue counterstain. Scale bars: (**a**,**c**–**e**) = 50 μm & (**b**) = 100 μm.

The basal membrane of the aggregate gland cells showed intense staining for CA (Fig. 11a). Cells in a small region between the distal end of the gland and the beginning of the duct had abundant small homogenous granules with strong black staining. The apical membrane of the cells in this region also showed intense staining for CA (Fig. 11b). The ducts of the aggregate glands showed variable intensity of CA staining. In some parts of the ducts, the cuticular intima as well as the outer membrane of the surrounding cells showed strong black staining (Fig. 11c), whereas in other parts of the ducts the staining was very weak (Fig. 11e). Interestingly, some parts of the ducts displayed varying staining intensity within a cross-section (Fig. 11d). The cells of the nodules surrounding the epithelium in all parts of the aggregate ducts showed remarkably intense staining in the cytoplasm as well as their basal membrane. The nuclei of epithelial cells surrounding the duct and the cells in the nodules were unstained.

**Figure 12:**
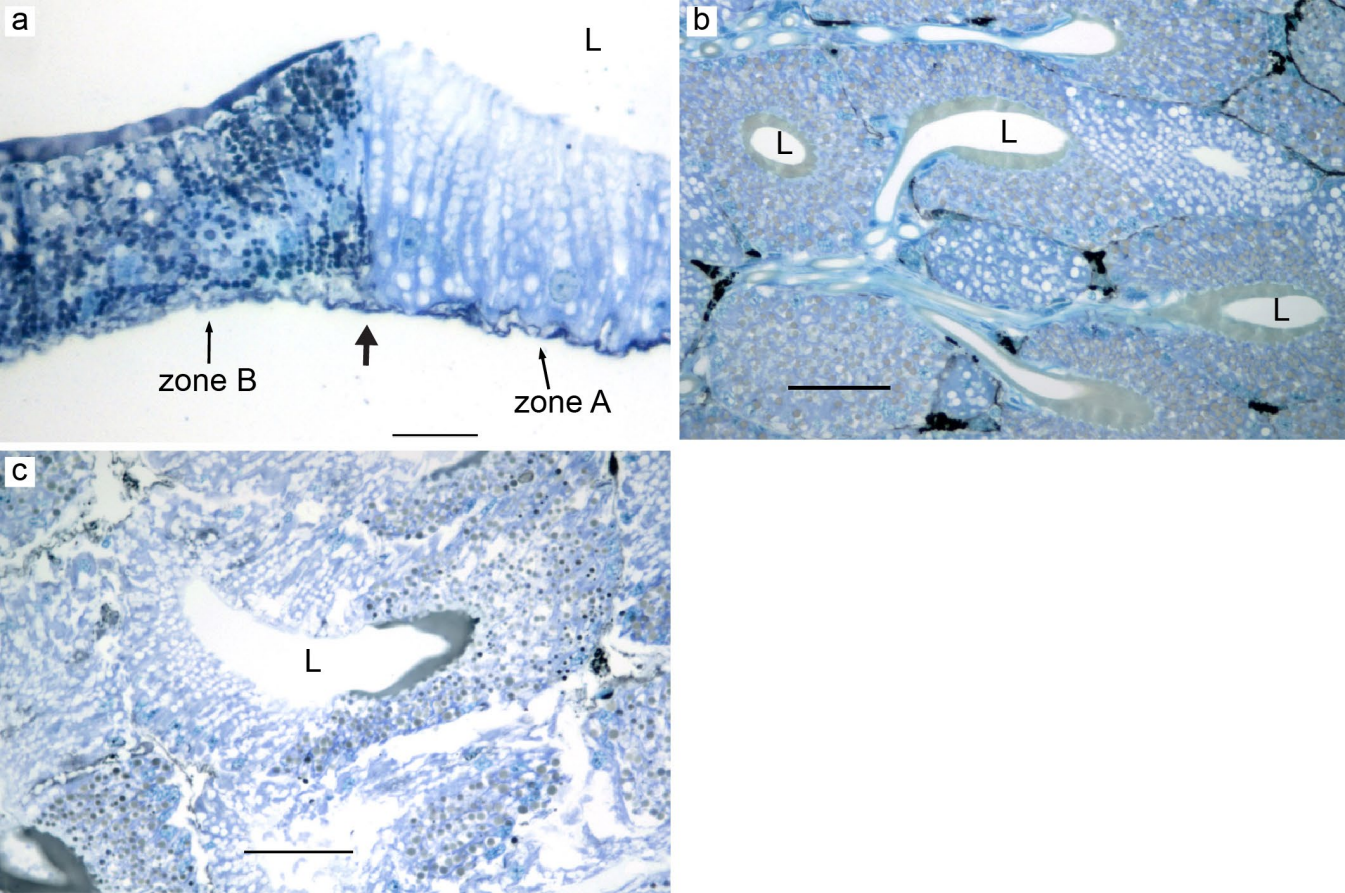
Carbonic anhydrase activity in flagelliform, piriform and aciniform glands of *L. sclopetarius*. (**a**) A small region in zone B of flagelliform glands show strong staining for CA in granules and cell membranes, arrow indicates border between zone A and B, L- lumen. (**b**) Weak CA staining in granules and apical lining of zone B cells of piriform glands. (**c**) Intermediate CA staining in some granules and the apical lining of Zone B cells of type II aciniform glands. Azure blue counterstain. Scale bars: (**a**–**c**) = 50 μm.

In the flagelliform gland, a few cells at the beginning of zone B had small, slightly irregular granules that stained intensely and some big granules that stained weakly for CA (Fig. 12a). The apical surface lining these cells showed abundant CA activity, while the basal membrane lining the whole flagelliform gland displayed weak CA staining.

In piriform glands, weak CA activity was seen in big granules of zone B cells (Fig. 12b) and in the secretion along the apical surface. No CA activity was detected in the cuticle of the duct. In zone B of type II aciniform glands*,* the CA activity was similar but slightly stronger as compared to piriform glands (Fig. 12c). These cells contained big granules that stained light grey as well as small granules that stained black. The secretion from zone B cells showed moderate CA activity.

### Thioflavin-S

**Figure 13:**
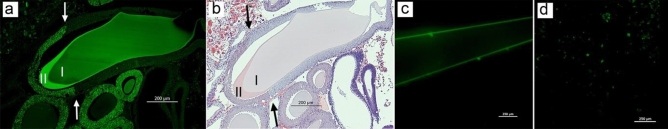
Thioflavin-S staining in major ampullate gland and silk fibers of *L. sclopetarius*. (**a**) Major ampullate gland showing green fluorescence in zone A & B cells. I & II indicate two layers distinguishable by Thioflavin-S. (**b**) Consecutive section stained with HE. I & II correspond to the layers detected in (**a**). In (**a**,**b**), arrows indicate the junction between zones B and C. (**c**) Native silk fibers stained with Thioflavin-S. (**d**) Aβ42 amyloid fibrils stained with Thioflavin-S served as a positive control. Scale bars: (**a**) = 200 μm, (**b**) = 100 μm, (**c**,**d**) = 250 μm.

Thioflavin is a common dye used to detect amyloid fibrils since it gives a characteristic fluorescence upon binding to the fibrillar aggregates^47^. The luminal contents and the content of the granules in the tail of the major ampullate gland showed bright green fluorescence. Strikingly, the fluorescence ceased at the junction of zone B and C, indicating that the granules of both zone A and B cells contain thioflavin-S positive components (Fig. 13a). We observed differential staining in the luminal contents that appeared similar to the granules of zone A and B cells (indicated as I & II in Fig. 13a,b). Other silk glands did not show any detectable fluorescence. Major ampullate silk fibers were also thioflavin-S positive, as anticipated (Fig. 13c). As expected, we observed thioflavin-S fluorescence in control amyloid fibrils made from the Aβ42 peptide that is associated with Alzheimer’s disease (Fig. 13d).

### Congo red

**Figure 14:**
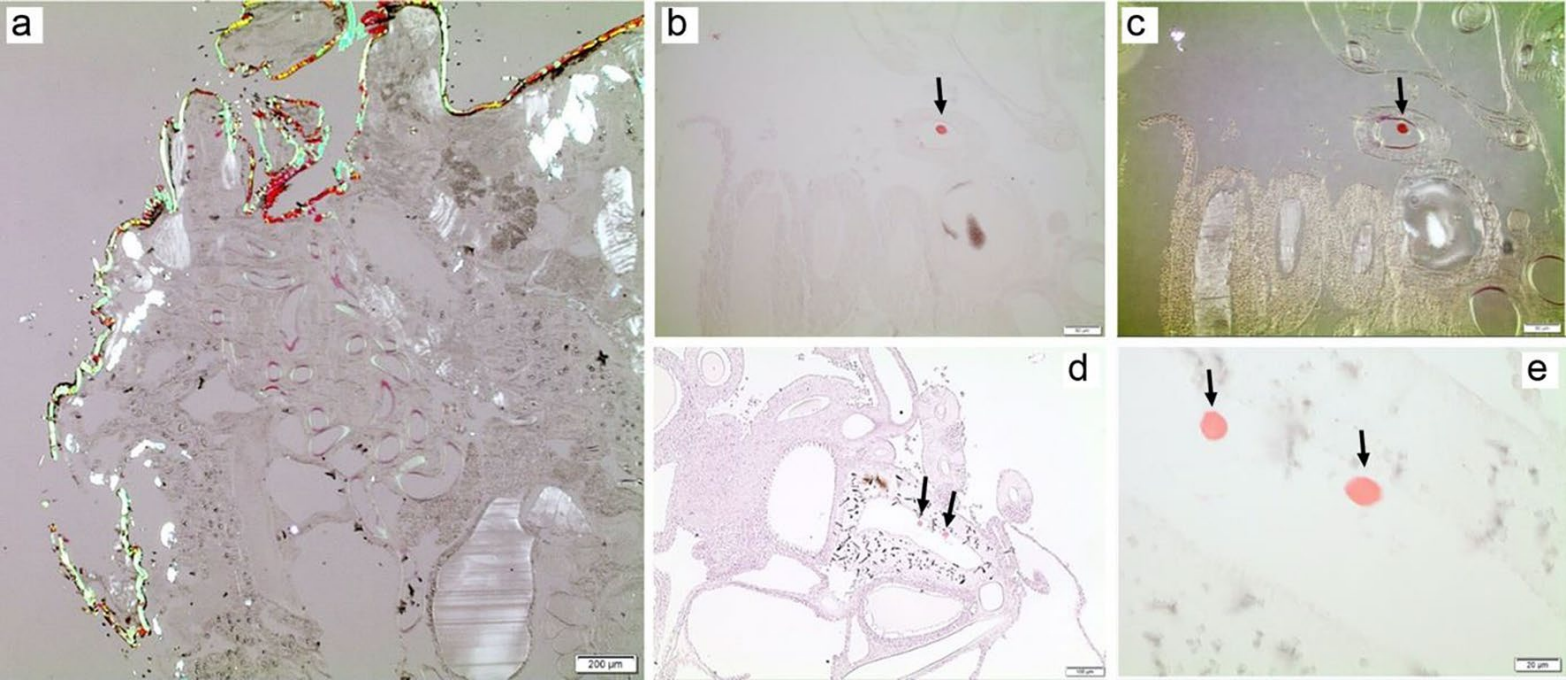
Congo red staining in silk glands of *L. sclopetarius.* (**a**) Part of a spider opisthosoma stained with Congo red and visualized in polarized light with crossed polars. A bright birefringence with predominantly green color is present in the exoskeleton. (**b**,**c**) A single strongly congophilic but not birefringent round structures found in one ampullate gland. (**b**) ordinary light, and (**c**) between crossed polars. (**d**,**e**) Two other congophilic structures, about 15 μm in diameter, visualized in ordinary light. At high magnification (**e**) there seems to be an outer demarcation of the structures. Scale bars: (**a**) = 200 μm, (**b**,**c**) = 50 μm, (**d**) = 100 μm, (**e**) = 20 μm.

Congo red staining and green birefringence are hallmarks of amyloid fibrils. Even without any counterstaining, the morphology of the opisthosoma was easy to examine due to a weak but evident greyish birefringence (Fig. 14). A weak Congo red affinity appeared in the lumen of ampullate glands close to the spinnerets (Fig. 14a). The walls and contents of the other glands showed no definite Congo red affinity but showed a grey birefringence between crossed polars. In one of the sections, a few round distinct structures appeared in a couple of tail cross-sections. They stained strongly with Congo red although no clear birefringence was seen (Fig. 14b–e). The exoskeleton showed a strong Congo red affinity and an equally strong green-yellow-orange birefringence.

### PAS

**Figure 15:**
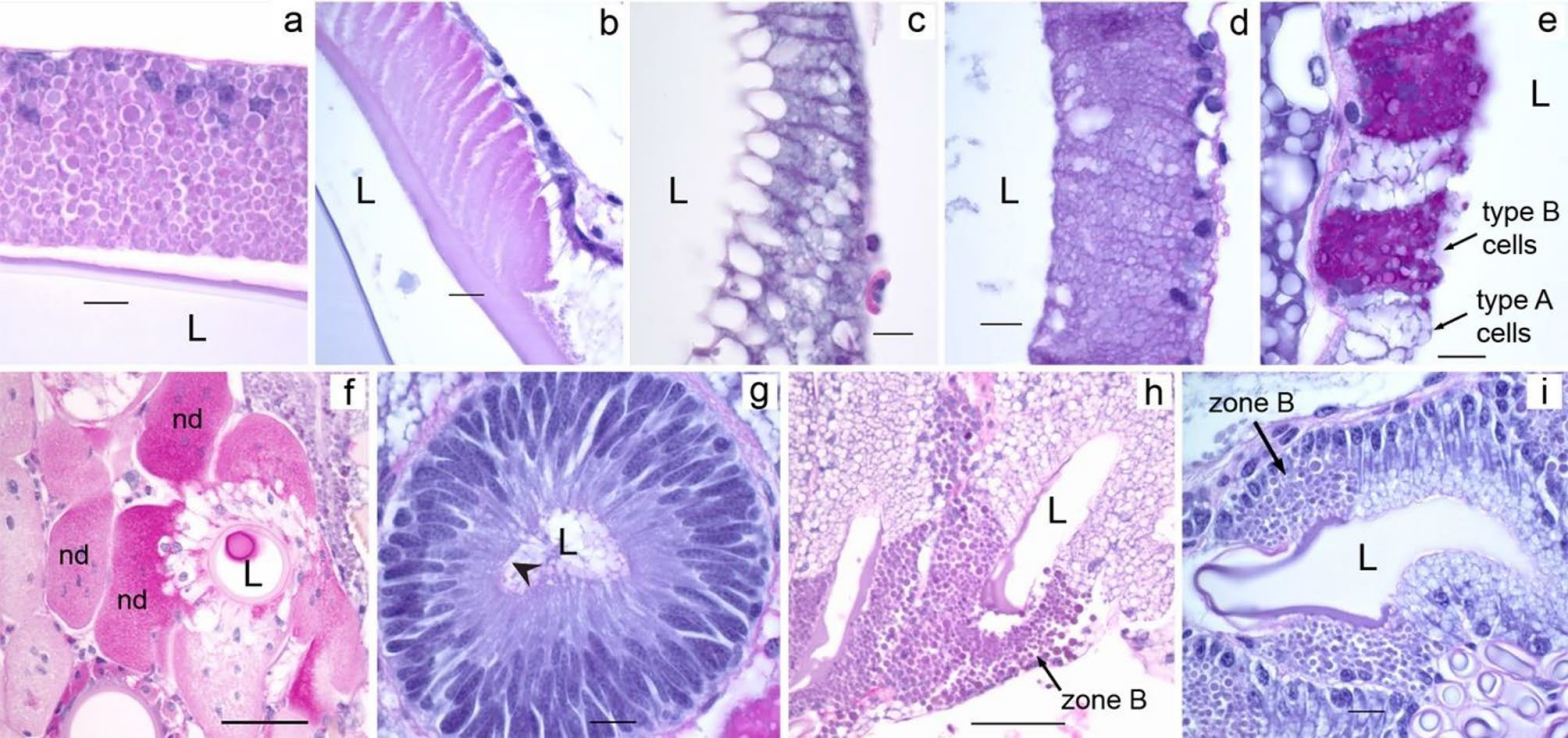
PAS staining in silk glands of *L. sclopetarius.* (**a**) PAS staining in granules of zone C cells of major ampullate gland. (**b**) Major ampullate funnel showing PAS staining. (**c**) Weak staining in zone A cells of flagelliform glands. (**d**) Intermediate staining in zone B cells of flagelliform glands. (**e**) Intense staining in type B cells of aggregate glands. (**f**) Nodular cells (nd) surrounding the aggregate ducts show intense PAS activity. (**g**) Weak staining in tubuliform cells apically (arrowhead). (**h**) Intermediate PAS staining in zone B cells of piriform glands. (**i**) Intermediate staining in zone B cells of type II aciniform glands, L – lumen. Scale bars: (**a**–**e**,**g**) = 10 μm, (**f**,**h**,**i**) = 50 μm.

PAS is a routinely used staining method to identify polysaccharides, mucosubstances, and mucins in tissue sections. In the ampullate glands, the zone C cells and the funnel stained weakly with PAS (Fig. 15a,b). In flagelliform glands, the zone A & zone B cells stained very weakly but differently (Fig. 15c,d). The staining was very intense in aggregate glands, especially, in the granules of type B cells (Fig. 15e) and in the nodules surrounding the ducts (Fig. 15f). Very weak staining was observed in the apical surface and the lumen of the tubuliform glands (Fig. 15g). Zone B cells of piriform glands were characterized by intermediate PAS staining (Fig. 15h). An intense PAS staining was observed in the granules and the apical lining of zone B cells of type II aciniform glands (Fig. 15i).

## Discussion

By using resin-embedded tissue, the morphology of all silk glands of *L. sclopetarius* could be visualized at a higher resolution compared to previously published work^5^. It allowed us to identify detailed morphological features including the presence of several cell types and distinct regionalization within all silk glands, some of which have not been described previously and that have implications for the architecture and composition of the silk fibers.

The major ampullate gland produces silk that is used by spiders as a lifeline as well as for the radial- and frame- lines of the web^15^. In agreement with previous work^11^, the epithelium of the tail and sac of the major ampullate gland was herein shown to be composed of three zones, A–C. We observed that the cells of each zone of the gland harbored numerous secretory granules which stained distinctly with HE. According to Andersson et al.^11^, zones A and B synthesize and secrete spidroins into the lumen, while the contents of zone C granules are unknown. In the present study, the contents secreted from each zone in the major ampullate gland did not mix, suggesting that the sequential addition of different components will lead to a complex and multi-layered structure of the fiber. In line with this, Sponner and coworkers demonstrated that the major ampullate silk has a multilayer arrangement, they identified four layers: the core, skin, glyco, and lipid layer^28^. Glycoproteins are most abundant in the glyco layer but can also be detected in the skin and the core layers. The function of the glyco and lipid layers is unknown, but they may aid in protecting against microbial degradation and may play a role in regulating the water content^24,28^. Here, we observed that the granules in zone C stained weakly for PAS, corroborating previous findings, and identifying the origin of the glycoproteins to be zone C epithelial secretions.

To detect morphological changes associated with increased protein production and silk secretion, we silked a group of spiders for 15 min before sacrifice and performed histological examination of the glands. However, we could see no difference in gland morphology or in CA activity compared to unsilked individuals. This is in contrast to a previous report^46^, but we speculate that the difference may be due to that we used a quite short silking time (15 min compared to 60 min in Moon & Tillinghast^46^), which may be a too short time frame for visible changes to occur.

Spider major ampullate silk has been suggested to be amyloid-like since parts of the spidroins in the fiber form β-sheets that resemble those of amyloid fibrils^21,48^. Before converting to mainly β-sheet structures, the spidroins are in unordered or helical conformations during synthesis in zone A and B cells and during storage in the lumen^49–51^. In an attempt to shed light on silk polymerization, we therefore used Congo red and thioflavin-S staining to locate amyloid-like structures in the major ampullate glands. The thioflavin-S stained sections showed fluorescence in all locations of the major ampullate gland where spidroins are expected to be found, and thus suggest that the spidroins in the granules of zone A and B and in the lumen have converted to β-sheet rich structures. This could possibly be a consequence of the treatment associated with the embedding of the tissues, but it is still surprising that only secretions from zone A and B cells in the major ampullate gland stained with thioflavin-S. The lack of staining in the vesicles of zone C cells further strengthens the notion that these cells do not secrete spidroins to a large extent. Congo red staining with concomitant birefringence under crossed polars is a more specific dye for amyloid than thioflavin-S, and a clear affinity of Congo red with typical birefringence is usually seen only after distinct amyloid fibrils have been formed. In this study, we could not detect distinct staining of any part of the gland in sections stained with Congo red. The strong affinity for the dye and concomitant birefringence of the exoskeleton likely depends on non-amyloid components.

The minor ampullate gland is similar in anatomy to the major ampullate and makes silk for the temporary scaffolding of the web^15^. To our knowledge, this is the first study which reports that the secretory portion (sac and tail) of the minor ampullate gland is composed of three cell types. The higher resolution of the thin (1 μm) resin sections made it possible to identify the transition between zone A and B compared to previous studies that have reported only two cell types in these glands^5,34^. Active CA was identified in zone C and funnel regions of the minor ampullate glands and zone C stained weakly for PAS. These results are very similar to major ampullate glands, and it is plausible to assume that minor and major ampullate silks are similar in structure.

Flagelliform glands produce silk for the capture spiral of the web^15^ and in this study, two zones containing histologically distinct cells were identified in these glands. Numerous irregular unstained granules were observed in the secretory portion of the gland, which may indicate that the content was dissolved during processing. The morphology of the flagelliform glands of this study is similar to what Mullen regards as median ampullate glands^5^. We noted that CA activity was localized to a short distinct region in the beginning of zone B, which may indicate that these cells should be regarded as a third cell type, but these cells could not be distinguished from the other cells in zone B when examined with HE. An interesting observation was that the CA activity was found in the middle of the gland and not in the distal part as in other glands producing silk fibers.

We identified two types of aciniform glands in *L. sclopetarius*. Aciniform silk is used for swathing insects and for the inner layer of egg cocoons^15^. It is not known if these glands produce two different types of silk fibers and if so, whether they are produced by the two different types of aciniform glands. According to Moon et al. type I glands contain electron-dense protein granules, whereas type II glands contain electron-lucent lipid granules in *Trichonephila clavata*^38^. In the current study, the two aciniform gland types both consisted of two cell types each confined to different zones. We could neither detect CA activity nor any PAS-positive structures in the type I glands. However, active CA was found in zone B cells of type II aciniform glands, which also stained strongly with PAS indicating the presence of carbohydrates/glycoproteins. This indicates that at least type II aciniform glands, which are more numerous than type I glands, may produce silk by a similar pH-dependent mechanism as the major ampullate glands.

Aggregate glands produce a viscous solution that covers the core fiber produced by flagelliform glands^52^. We demonstrate, for the first time, that aggregate glands have two types of cells (A and B). The two cell types differed in height and their staining of granules. The different cell types were not confined to specific zones, but type B cells were more abundant than A cells. The aggregate ducts were convoluted and surrounded by several nodules. The ducts of two aggregate glands were arranged in a triad with one flagelliform duct in the center, as previously seen in two other species^12,37, 53^. The triad arrangement of these ducts is thought to facilitate the extrusion of the silk so that the axial fibers formed by flagelliform glands are simultaneously covered with glue droplets produced by aggregate glands as the silk emerges out^54^. In the current study, the strongest staining for both CA and PAS was detected in the ducts of the aggregate glands, in the cytoplasm of the nodular cells, and in parts of the cuticular intima. In accordance, ultrastructural studies have shown that the nodular cells are rich in glycogen and mitochondria^55^, and the internal cells of the ducts have characteristics of absorbing cells^56^. In two orb-weaving spiders, the nodules are shown to have glycogen phosphorylase, an enzyme vital for glycogenolysis^57^. Some studies suggest that these cells transport water, ions (especially phosphate), and possibly some organic compounds^58^ using glycogen as energy^57^, providing a possible explanation for how the viscous solution is produced. Additionally, it’s worth noting that aggregate proteins are known to undergo post-translational glycosylation, which could also potentially account for the observed intense PAS staining in these glands^59–63^.

In this study, all three pairs of tubuliform glands had identical morphology, consisted of only one cell type, and had a funnel that connected the gland and the duct as previously reported by^5^. The role of the funnel is not clearly understood, but it is seen only in the ampullate and tubuliform glands. The pink spiral structures observed in the epithelial cells of these glands (Fig. 6b) could be endoplasmic reticula which are reported to be abundant in these glands^64,65^. We did not detect CA activity in the tubuliform glands of *L. sclopetarius*, which is in contrast to the findings in other orb weavers^21^. Tubuliform silk is used for making the egg sack and is therefore only active during a phase in the spider´s life. Accordingly, the morphology of the gland varies with the development of oviducts and eggs^64^. Evidence for different developmental stages of the tubuliform glands was seen in our sections but we could not determine if this was the reason that no CA activity could be detected.

Piriform silk is used for cementing the junctions in the web as well as for making the attachment discs that secure the ampullate fibers to the substrate^15^. Results of the current study showed that these glands were composed of two zones with distinct cell types as described previously in *L. sclopetarius*^5^ and *A. diadematus*^31^. Active CA and PAS-positive structures were detected in zone B cells. The PAS structures might be glycoproteins which are thought to serve an adhesive function to the silk equivalent to that of sericin^31^. The silk produced by piriform glands is composed of many small fibers which may contribute to the overall mechanical properties of the attachment discs^66^. The polymerization of these silk fibers may be aided by acidic environments similar to that in major ampullate glands which can be facilitated by active CA in these glands.

In the major ampullate gland, CA is responsible for generating and upholding a pH gradient, from 7.6 to < 5.7^21^. This pH gradient is important for major ampullate spidroin assembly and phase transition in the duct. It is the spidroin terminal domains that are primarily affected by the increasingly acidic conditions and mediate fiber formation^20,21, 67–70^. Recent studies of the spider genome and transcriptome in the silk glands show that the terminal domains are present in all spidroin types^71–76^. This, combined with our findings that CA activity is found in all silk glands (except the tubuliform & type I aciniform glands) suggests that the pH gradient may be universal to silk glands and that polymerization of all different types of spider silk is dependent on a pH gradient generated by CA. However, the location of CA in the flagelliform glands is somewhat odd and how this relates to a possible pH gradient in these glands requires further studies. Regarding the lack of CA activity in the tubuliform gland, it should be noted that another study has identified CA activity also in this gland^21^. As mentioned earlier, the tubuliform gland is active only in association with egg laying and construction of the egg sack, which could be true also for the aciniform type I glands. It is not established which of the aciniform glands that are responsible for making the inner lining of the egg sack but provided that aciniform type I glands produce this silk, a plausible explanation to the lack of detectable CA activity could be that both glands are dormant in our sections. In the aggregate glands, strong staining for CA was detected in the ducts, in the cytoplasm of the nodular cells, and in parts of the cuticular intima. We speculate that the abundant CA activity seen in these cells plays an active role in generating the bicarbonates or protons needed for glue formation.

In the α-CA class, 16 CA isozymes have been identified to date which are subdivided into the following groups: cytosolic, mitochondrial, secreted, GPI-anchored, transmembrane, and acatalytic^77^. Genes coding for two different orthologs (isozymes) of carbonic anhydrases (Ca13 and Ca14) have been identified as silk gland-specific transcripts in *Trichonephila clavipes*^78^. CA13 and CA14 belong to the cytosolic and transmembrane (extracellular active site) classes, respectively. Based on the staining in our sections, the CA activity is associated with cell membranes, granules, and/or cytoplasm, but it is beyond the scope of this study to assign the spider CAs to specific classes. We detected active CA in several other structures in the opisthosoma besides silk glands such as hemolymph, muscles, book lungs, and oviducts (results not shown). Except for the importance of CA to generate a pH gradient in the major ampullate gland, little is known about spider-specific CAs and their functions.

In conclusion, we have characterized all seven silk glands and ducts of *L. sclopetarius* using various morphological methods. The regionalization of multiple cell types in all glands, except tubuliform and aggregate glands, suggests that the secretions may be added in layers, indicating that spider silk fibers are complex layered structures. Based on the identification of different cell types, the addition of secretory products in layers, and the localization of active CA we speculate that the process of silk formation in minor ampullate, aciniform type II, and piriform glands may be similar to the mechanism previously described for major ampullate glands. In addition, PAS-positive structures in several glands support previous findings that carbohydrates are components of silk fibers.

## Materials and methods

Adult females (n = 32) of *L. sclopetarius* were collected in Uppsala, Sweden between June and October of 2019. They were kept in individual containers, fed small crickets and water.

### Tissue processing and light microscopy

Spiders were anesthetized using CO_2_ gas and severed at the pedicel. One group of spiders (n = 7) was silked for about 15 min. To make the spiders spin major ampullate silk, they were gently made to fall several times from a wooden block on which the silk was collected, before they were sacrificed. The dissection was performed on a wax plate placed on ice using 9 mg/mL (154 mM) sodium chloride (Fresenius Kabi AG, Germany) with the help of a Leica M60 stereomicroscope equipped with a Leica IC80 HD camera. Individual glands and whole opisthosomas were fixed in 2.5% glutaraldehyde in 67 mM phosphate buffer, pH 7.2 for 24 h at 4 °C, and later rinsed in 67 mM phosphate buffer. The tissues were dehydrated in increasing concentrations of ethanol (50, 70, 90, and 100%, for 30 min each), infiltrated, and embedded in a water-soluble glycol methacrylate (Leica Historesin). Sections (1 and 2 μm) were cut with glass knives using a Leica RM 2165 microtome, stained with hematoxylin–eosin (HE), and mounted using Agar 100 resin. HE staining is one of the most common techniques used in histological analysis that allows for visualization of cells and tissues, with hematoxylin staining cell nuclei blue-purple and eosin staining cytoplasm and extracellular matrix pink.

### Staining for carbonic anhydrase activity

CA activity was detected using a very selective histochemical method^45^. Serial sections were incubated floating on a solution containing 156.6 mM NaHCO_3_, 3.5 mM CoSO_4_, 52.6 mM H_2_SO_4_, and 11.7 mM KH_2_PO_4_. In locations with active CA, a cobalt-phosphate-carbonate complex was formed. Sections were moved to a solution containing CoSO_4_ and (NH_4_)_2_S, and the complex was converted to a black cobalt sulphide precipitate. The sections were counterstained with azure blue. At least one section from each series was incubated with the CA inhibitor acetazolamide, to identify areas with unspecific staining. To check that the CO_2_ anesthesia did not affect CA activity, a few spiders were euthanized using low temperature (− 20 °C, 5 min). Sections from these spiders were otherwise treated as above. All resin sections were evaluated using a Nikon Microphot-FXA (Tekno Optik AB) microscope equipped with a Nikon FX-35DX camera. Images were captured and edited using the software Eclipse Net v1.20.0.

### Staining with thioflavin-S

Whole opisthosomas of six spiders were fixed in 4% formalin for 24 h at room temperature then rinsed in 67 mM phosphate buffer and embedded in paraffin. The samples were sectioned (4 μm) using a MICROM HM 355S microtome. For staining with thioflavin-S, deparaffinized sections were incubated in 0.1% thioflavin-S solution (prepared in 50% ethanol) for 1 h at room temperature (protected from light). The sections were washed in 80%, 95% ethanol, and pure water, respectively, dried, and mounted with coverslips. Native silk fibers collected from bridge spiders were secured on glass slides and incubated with 0.1% thioflavin-S solution for one hour at room temperature. Amyloid fibrils prepared from 24 μM Aβ42 monomers, which were recombinantly produced as described in Ref.^79^, were used as positive control whereas silk fibers incubated in 50% ethanol were used as negative controls. Sections and fibers were evaluated using a Nikon Eclipse E600 fluorescence microscope equipped with a Nikon DXM1200 digital camera.

### Staining with congo red and PAS

For Congo red staining, paraffin sections were stained with alkaline Congo red^80^, modified as described in Westermark^81^, without counterstain and examined, with and without crossed polars, in an Olympus BX51 polarization microscope equipped with a U-AN350P polarization device. For PAS, deparaffinized sections were incubated in 0.5% periodic acid (5 min), rinsed in distilled water, incubated in Schiff’s reagent (20 min, protected from light), and rinsed in tap water. The sections were counter-stained with Mayer’s hematoxylin for 1 min and mounted. Sections were evaluated using the Nikon Microphot-FXA (Tekno Optik AB) microscope equipped with Nikon FX-35DX camera. Images were captured and edited using the software Eclipse Net v1.20.0.

### Supplementary Information

Supplementary Figures.

## Data Availability

The data from this study are available upon request from the corresponding author.
