# Supplementary material for: Regionalization of cell types in silk glands of Larinioides sclopetarius suggest that spider silk fibers are complex layered structures
Source: Sci Rep. 2023 Dec 14;13:22273. doi: 10.1038/s41598-023-49587-z (PMC10721825; doi:10.1038/s41598-023-49587-z)
Supplement: Supplementary file 1 — Supplementary Figures. [file 41598_2023_49587_MOESM1_ESM.docx]

Regionalization of cell types in silk glands of Larinioides sclopetarius suggest that spider silk fibers are complex layered structures

Scientific Reports

**Author information**

Sumalata Sonavane^1^, Per Westermark^2^, Anna Rising^1,3^, Lena Holm^1*^

^1^ Department of Anatomy, Physiology and Biochemistry, Swedish University of Agricultural Sciences, Uppsala, Sweden

^2^ Department of Immunology, Genetics and Pathology, Uppsala University, Uppsala, Sweden

^3^ Department of Biosciences and Nutrition, Karolinska Institutet, Neo, Huddinge, Sweden

*Corresponding author ([lena.holm@slu.se](mailto:lena.holm@slu.se))

**Supplementary Figures**


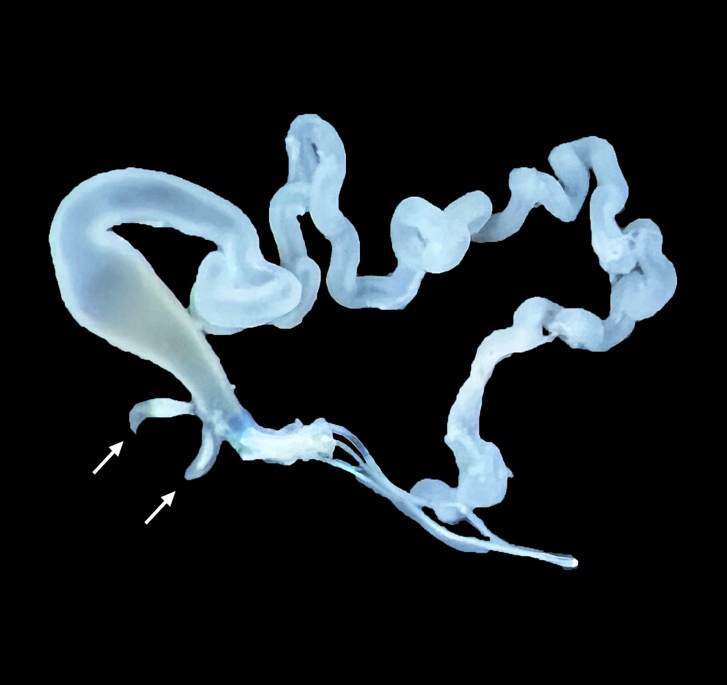


**SI Fig 1** One pair of secondary major ampullate glands (indicated with arrows) is attached to the duct of each primary major ampullate gland


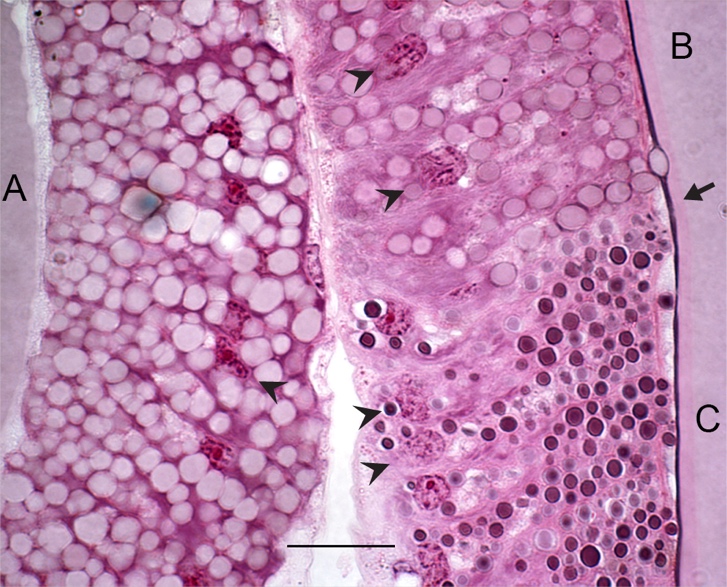


**SI Fig 2** Three zones (A–C) of major the ampullate glands. Arrow indicates the sharp transition from zone B to zone C cells, arrowheads show the basally located nuclei. HE, Scale bar = 20 μm


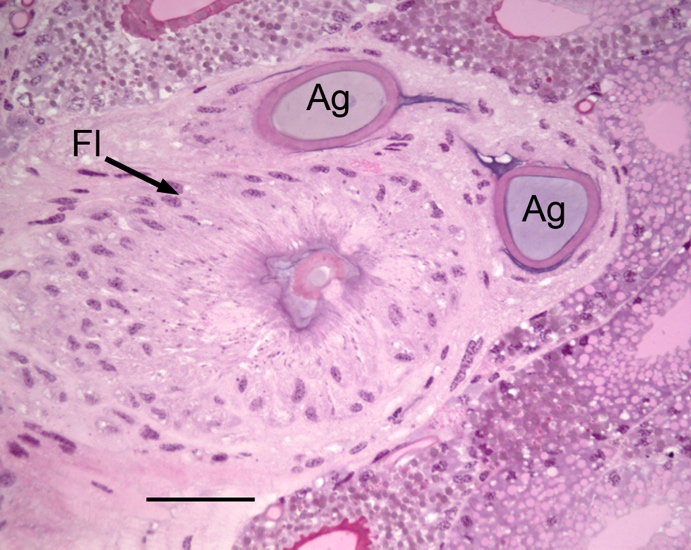


**SI Fig 3** The triad formed by two aggregate ducts (Ag) and one flagelliform duct (Fl). Note the similar diameters of aggregate ducts. HE, Scale bar = 20 μm
